# Comprehensive exploratory autoantibody profiling in patients with early rheumatoid arthritis treated with methotrexate or tocilizumab

**DOI:** 10.1371/journal.pone.0241189

**Published:** 2020-12-10

**Authors:** Xavier M. Teitsma, Jenny Devenport, Johannes W. G. Jacobs, Attila Pethö-Schramm, Michelle E. A. Borm, Petra Budde, Johannes W. J. Bijlsma, Floris P. J. G. Lafeber

**Affiliations:** 1 Department of Rheumatology and Clinical Immunology, University Medical Center Utrecht, Utrecht University, Utrecht, Netherlands; 2 Department of Rheumatology & Clinical Immunology, Utrecht University, Utrecht, Netherlands; 3 Pharmaceuticals Division, F. Hoffmann-La Roche, Basel, Switzerland; 4 Roche Nederland BV, Woerden, Netherlands; 5 Department of Medical Research, Oncimmune Germany GmbH, Dortmund, Germany; Nippon Medical School, JAPAN

## Abstract

**Background:**

We sought to identify immunoglobin G autoantibodies predictive of early treatment response to methotrexate, the recommended first-line therapy for patients with newly diagnosed rheumatoid arthritis, and to the interleukin-6 receptor inhibitor biologic tocilizumab, initiated as the first disease-modifying anti-rheumatic drug.

**Materials and methods:**

In baseline sera of a subset of patients with newly diagnosed rheumatoid arthritis in the U-Act-Early study, selected based on specific responder/non-responder criteria using the Disease Activity Score assessing 28 joints (DAS28) within the first 20 weeks, we measured immunoglobin G antibody reactivity against 463 protein antigens and performed supervised cluster analysis to identify predictive autoantibodies for treatment response. The analysis subset comprised 56 patients in the methotrexate arm (22 responders, 34 non-responders) and 50 patients in the tocilizumab arm (34 responders, 16 non-responders). For comparison, these analyses were also performed in 50 age- and gender-matched healthy controls.

**Results:**

Increased reactivity in responders versus non-responders was found in the methotrexate arm against two antigens—DOT1-like histone lysine methyltransferase (p = 0.009) and tropomyosin (p = 0.003)—and in the tocilizumab arm against one antigen—neuro-oncological ventral antigen 2 (p = 0.039). Decreased reactivity was detected against two antigens in the methotrexate arm—G_1_ to S phase transition 2 (p = 0.023) and the zinc finger protein ZPR1 (p = 0.021). Reactivity against the identified antigens was not statistically significant in either treatment arm for patients with rheumatoid factor–positive versus–negative or anti-cyclic citrullinated test–positive versus test–negative rheumatoid arthritis (p ≥ 0.06).

**Conclusions:**

Comprehensive profiling of baseline sera revealed several novel immunoglobin G autoantibodies associated with early treatment response to methotrexate and to tocilizumab in disease-modifying anti-rheumatic drug-naive patients with rheumatoid arthritis. These findings could eventually yield clinically relevant predictive markers, if corroborated in different patient cohorts, and may facilitate future benefit in personalised healthcare.

## Introduction

Rheumatoid arthritis (RA) is a chronic autoimmune disease characterised by inflammation and primarily affecting small joints of hands and feet [1]. Generally, the disease is associated with serological markers of systemic autoimmunity, marked by elevated titres of autoantibodies in serum or synovial fluid. Although several autoantibodies are frequently detectable in more than one immune disease, rheumatoid factors (RF), particularly anti-citrullinated protein antibodies (ACPA), are specific for RA; tests for these biomarkers are used in clinical practice for their diagnostic and, to a lesser extent, prognostic value. Approximately 60% to 90% of RA patients have positive RF and/or positive results of ACPA tests [2]. It has been demonstrated that the production of these antibodies can precede the development of clinical manifestations of RA by years [3] and that these antibodies are associated with a less favourable outcome, such as more erosive disease [4]. Patients with RF-positive or ACPA-positive RA have higher levels of inflammation and less frequently achieve remission [5, 6]. To what extent these autoantibodies can influence treatment decisions remains to be fully elucidated, though several studies suggest that RA patients with higher levels of RF or ACPA especially benefit from rituximab, a B cell–depleting therapy [7, 8].

The complex pathophysiology of RA cannot fully be captured by either of these antibodies (RF or ACPA) alone because not all patients have RF-positive or ACPA-positive disease, and it has been shown that other autoantibodies, such as anti-carbamylated protein antibody, are involved [9, 10], which also demonstrates the active role of antibody-mediated (i.e. humoural) autoimmunity. Among RA patients, a broader autoantibody profile, characterised by a greater variety of immunoglobin isotopes, is associated with better early treatment response to a methotrexate-based strategy [11]. Identifying new autoantibodies would therefore be highly interesting because these might be involved in the pathophysiology of RA and could be associated with treatment outcomes. In this regard, the treatment response to methotrexate is worth investigating because of international recommendations/guidelines to use methotrexate as first-line and anchor therapy in RA [12, 13].

The aim of this exploratory analysis was to identify immunoglobin G (IgG) autoantibodies associated with early treatment response to methotrexate or to tocilizumab (*i*.*e*. interleukin-6 [IL-6] receptor inhibitor), each initiated as a first-line disease-modifying anti-rheumatic drug (DMARD) in the U-Act-Early study, in baseline serum samples from patients with early RA. The ultimate goal would be to develop a set of predictive biomarkers to identify RA patients who are likely to have an unfavourable treatment response to specific individual DMARDs and are therefore at a higher risk for erosive disease [14–16], facilitating personalised therapy in patients with newly diagnosed RA who are commencing therapy.

## Materials and methods

### Study design

This analysis included patients who were diagnosed with very early RA and who participated in the 2-year, multi-centre, double-blind, placebo-controlled, three-arm randomised U-Act-Early strategy trial (ClinicalTrials.gov, NCT01034137). This treat-to-target strategy trial enrolled 317 DMARD-naïve patients with newly diagnosed RA. Adults meeting the 1987/2010 RA classification criteria [17, 18] with disease activity (disease activity score assessing 28 joints (DAS28) >2.6) and a symptom duration of ≤1 year were eligible. The study goal was to achieve sustained remission, defined as DAS28 <2.6 with ≤4 swollen joints for at least 24 weeks, with initial therapy including methotrexate, or tocilizumab, or methotrexate plus tocilizumab. Methotrexate (oral) was started at 10 mg/week and was increased every 4 weeks in 5-mg increments (maximum, 30 mg/week) until remission or maximum tolerable dose; tocilizumab was given intravenously every 4 weeks at a fixed dose of 8 mg/kg (maximum, 800 mg per dose), as described previously [19]. If the treatment target—sustained remission—was not achieved with these regimens, hydroxychloroquine (200 mg twice per day orally) was added to the initial treatment regimen. Thereafter, if remission still was not achieved, patients switched to another treatment regimen: those who initially started with methotrexate or tocilizumab therapy switched to methotrexate plus tocilizumab therapy, and those who received the combination therapy switched to the standard of care therapy, usually including a tumour necrosis factor inhibitor.

The Medical Ethics Committee of the University Medical Center Utrecht approved the U-Act-Early study for all participating hospitals. All patients signed informed consent before study entry.

### Patient selection

To identify autoantibodies associated with early treatment response to methotrexate or tocilizumab, patients classified as responders and non-responders from both monotherapy arms of the U-Act-Early study were selected for inclusion. Patients were considered responders if they were categorised in the lowest tertile of average DAS28 from week 4 until week 20 (i.e. excluding pre-treatment/baseline DAS28), in remission (DAS28 <2.6) at week 20 and in remission during ≥2 visits within the first 20 weeks. Patients were considered non-responders if they were categorised in the highest tertile of average DAS28 from week 4 until week 20, were not in remission during two consecutive visits within the first 20 weeks and experienced <1.2 decline in DAS28 after 20 weeks. Patients who were not considered responders or non-responders were excluded from the analyses. In addition, 50 age- and gender-matched healthy controls were included for comparison.

### Autoantibody profiling

A comprehensive multiplex profiling technique was used to measure baseline serum IgG antibodies against 463 human protein antigens, consisting of well-known and novel antigens identified in high-content profiling studies in rheumatic diseases [20–22]. The antigen array content was designed as described [23] to include 45 well-known diagnostic rheumatic disease antigens and proteins based on their potential relevance to pathogenic pathways in autoimmune diseases such as cytokines, cytokine receptors, chemokines, interferons and matrix metalloproteinases. The full list of antigens is provided in S1 File. The bead-based assay (MagPlex microspheres; Luminex Corporation, Austin, TX, USA) used in the present study has been shown to have good overall agreement with the conventional enzyme-linked immunosorbent assay [24]. In addition, serum samples from 50 age- and gender-matched healthy controls were measured simultaneously for comparison. Antigens were purchased from Diarect AG (Freiburg, Germany) and Merck KGaA (Darmstadt, Germany) or were produced in-house using *Escherichia coli* SCS1 carrying plasmid pSE111, containing an N-terminally located hexa-histidine-tag. Antigens were affinity-purified under denaturing conditions with the use of funnel columns (Protino Ni-IDA 1000; Macherey-Nagel, Düren, Germany) and were fixed to magnetic carboxylated color-coded beads (Luminex Corporation). The manufacturers’ protocols were adapted to enable multiplexing using semi-automated procedures. Beads were resuspended in 120 μl activation buffer and activated by adding 15 μl 1-ethyl-3-(3-dimethylaminopropyl) carbodiimide (50 mg/ml) and 15 μl N-hydroxysuccinimide (50 mg/ml) and then washed three times. Coupling of antigens was performed for 2 hours at room temperature; coupled beads were washed and resuspended in 100 μl blocking buffer. Finally, beads were combined and stored at 4°C to 8°C until use. Forty-one protein antigens were citrullinated using peptidyl arginine deiminase from rabbit skeletal muscle (PADR; Merck KGaA). In total, 50,000 antigen-coupled beads were incubated with 12.5 mU PADR for 4 hours at 37°C in a reaction buffer, followed by three washing steps. Serum samples were diluted 1:100 in assay buffer (Candor Biosciences, Wangen, Germany), added to the bead mix and incubated for 20 hours at 4°C to 8°C. After washing, the beads were incubated with 5 μg/ml R-phycoerythrin-labelled detection antibody (Dianova, Hamburg, Germany) for one hour at room temperature. The beads were then washed and resuspended in 100 μl sheath fluid (Luminex Corporation) and thereafter analysed in a multiplexing platform (FlexMap3D; Luminex Corporation). IgG reactivity values are given as median fluorescence intensity (MFI), and data of antigens fulfilling the minimum bead count criterion (>10 beads measured per bead ID) were used for analysis. The readout range for the MFI was confirmed with positive control sera as well as with anti-HIS6 antibodies. To monitor the inter-assay coefficient of variation (CV), in-process control samples were measured in triplicate on each 96-well serum plate using the autoantibody MFI values against all measured antigens. The mean inter-plate and intra-plate CV of this screen was 11% and 10%, respectively.

### Statistical analysis

Baseline clinical demographics are described as mean (standard deviation [SD]), median (interquartile range) or proportion (%). The mean change in disease activity, measured by DAS28, from baseline to week 20 was assessed in each strategy arm using a linear mixed model with random intercept and baseline DAS28, week of measurement and group (responders vs non-responders) as fixed effects. Antibody concentrations, expressed by MFI, were normalised (natural log transformed) and thereafter standardised (*z*-scores).

Candidate antigens in each treatment arm were identified in sequential steps, first by performing partial least square discriminant analysis, a supervised cluster technique with group (responders vs non-responders) as binary classifier and individual antibodies (transformed and standardised as stated) as predictors; antibodies with variable importance on projection score ≥2 and p < 0.05 were selected for further analyses. Next, logistic regression analysis with manual stepwise backward selection (cutoff, p < 0.05) and group (responders vs non-responders) as the dependent variable was performed to identify, within each treatment arm, the most relevant antigens. The discriminative performance of these antigens was evaluated by the area under the receiver operator characteristics curve (AUROC), considered ‘excellent’ (0.9–1.0), ‘good’ (0.8–0.9), ‘fair’ (0.7–0.8), ‘poor’ (0.6–0.7) or ‘fail’ (<0.6), for curves plotted above the reference line [25]. In addition, concentrations of the identified antigens were compared with concentrations measured in the matched healthy controls using logistic regression, with controls versus patients as the dependent variable.

To evaluate the additive value of antigens to clinical parameters in the prediction of early treatment response to methotrexate or tocilizumab, we assessed the following patient characteristics and clinical baseline variables: age, gender, body mass index, current smoker (yes/no), alcohol consumption (yes/no), symptom duration, rheumatoid factor (positive/negative), anti-cyclic citrullinated peptide (positive/negative), C-reactive protein, DAS28, Health Assessment Questionnaire score and Sharp/van der Heijde score. Erythrocyte sedimentation rate (ESR) was not separately assessed because DAS28 was calculated using ESR. Univariably selected clinical predictors (cutoff, p ≤ 0.15) were included in the multivariable regression model, with group (responders vs non-responders) as the dependent variable, and the number of predictors was further reduced by backward manual selection, deleting, step-by-step, those with the highest p value, until all p values were ≤0.10. The more lenient cutoff for clinical predictors, in contrast to that for the antigens, was applied because of the lower number of clinical parameters compared with the number of antigens investigated. Finally, the AUROCs of the clinical versus the antigen model and versus the combined model (i.e. clinical plus antigen) were tested for significance using DeLong’s test [26]. All statistical analyses were performed using R version 3.4.3. (R Foundation for Statistical Computing, Vienna, Austria).

## Results

### Clinical evaluation

**Fig 1:**
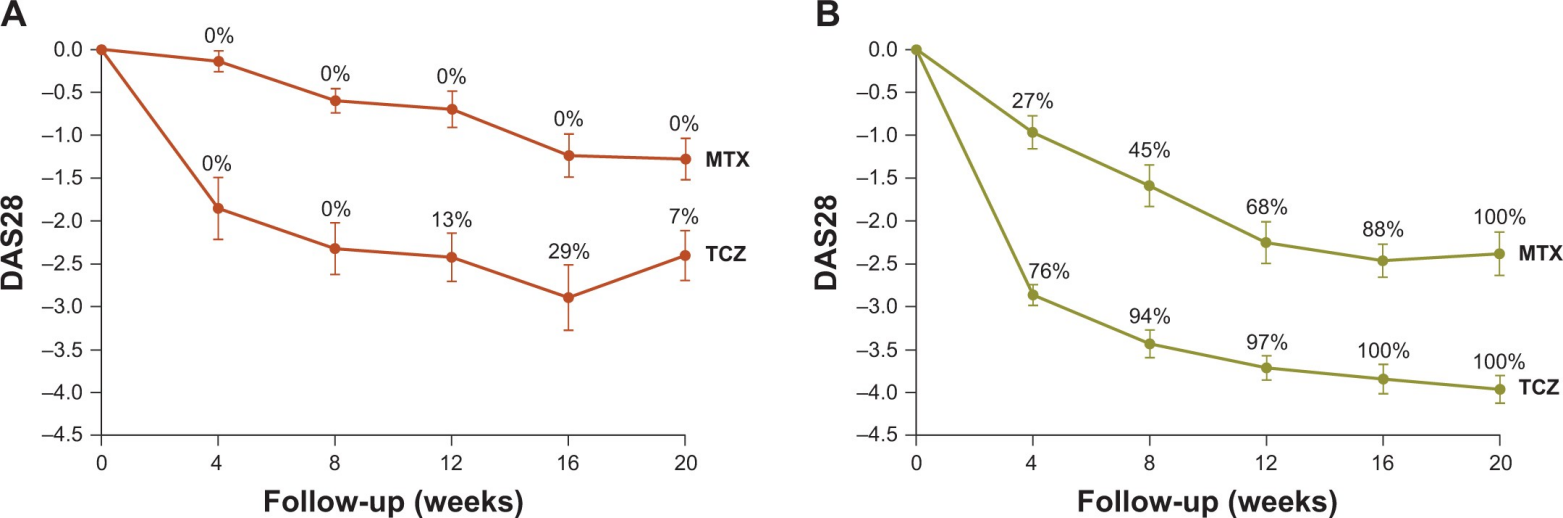
Mean (SE) change in disease activity from baseline over time of patients selected from U-Act-Early. (**A**) Non-responders from the methotrexate (n = 34) and tocilizumab (n = 16) arms and (**B**) responders from the methotrexate (n = 22) and tocilizumab (n = 34) arms. *Red* line depicts non-responders and *green* line depicts responders, with proportions (%) of patients in DAS28 remission (<2.6) indicated at each time point. DAS28, Disease Activity Score assessing 28 joints; MTX, methotrexate; SE, standard error of the mean; TCZ, tocilizumab.

Baseline demographics of the groups are shown in Table 1. In the methotrexate arm, 22 of 56 (39%) patients were classified as responders and 34 of 56 (61%) as non-responders; for the tocilizumab arm, these numbers were 34 of 50 (68%) and 16 of 50 (32%), respectively. When evaluating the mean change in disease activity score over time, corrected for baseline DAS28, indeed a statistically significantly lower DAS28 was found in both strategy arms for the responders (methotrexate arm: mean [95% CI] –1.88 [–2.20, –1.56], p < 0.001; tocilizumab arm: mean [95% CI] –1.72 [–1.93, –1.50], p < 0.001) compared with the non-responders. Furthermore, mean changes in DAS28 during follow-up were consistently lower in the tocilizumab arm than in the methotrexate arm for both non-responders (Fig 1A) and responders (Fig 1B). In the methotrexate arm, no patient classified as a non-responder achieved DAS28 remission (<2.6) at any time during the first 20 weeks; in the tocilizumab arm, this proportion was 29%.

**Table 1:** Baseline clinical characteristics of the patients selected from two U-Act-Early study arms.

	Methotrexate arm		Tocilizumab arm	
	Responders	Non-responders	Responders	Non-responders
	n = 22	n = 34	n = 34	n = 16
**Female gender, n (%)**	14 (64)	24 (71)	25 (74)	14 (88)
**BMI, kg/m**^**2**^**, mean (SD)**	25 (4)	27 (5)	25 (3)	27 (6)
**Age, years, mean (SD)**	49 (11)	53 (15)	52 (14)	54 (18)
**Caucasian, n (%)**	22 (100)	32 (94)	34 (100)	14 (88)
**Disease duration, days, median (IQR)**	29 (18–40)	25 (15–47)	27 (20–48)	27 (23–35)
**Current smoker, n (%)**	6 (27)	10 (29)	5 (15)	5 (31)
**RF positive, n (%)**	19 (86)	27 (79)	23 (68)	8 (50)
**Anti-CCP positive, n (%)**	19 (86)	25 (73)	23 (68)	9 (56)
**DAS28, mean (SD)**	4.4 (0.9)	6.0 (1.0)	5.0 (0.9)	5.9 (1.2)
**ESR, mm/h, median (IQR)**	40 (14–48)	24 (15–41)	21 (10–40)	36 (20–73)
**CRP, mg/L, median (IQR)**	20 (6–35)	9 (4–16)	8 (4–19)	15 (8–38)
**HAQ, mean (SD)**	1.2 (0.7)	1.0 (0.7)	1.1 (0.5)	1.3 (0.7)

BMI, body mass index; CCP, cyclic citrullinated peptide; CRP, C-reactive protein; DAS28, Disease Activity Score assessing 28 joints; ESR, erythrocyte sedimentation rate; IQR, interquartile range; RF, rheumatoid factor; SD, standard deviation.

### Candidate antigens

**Fig 2:**
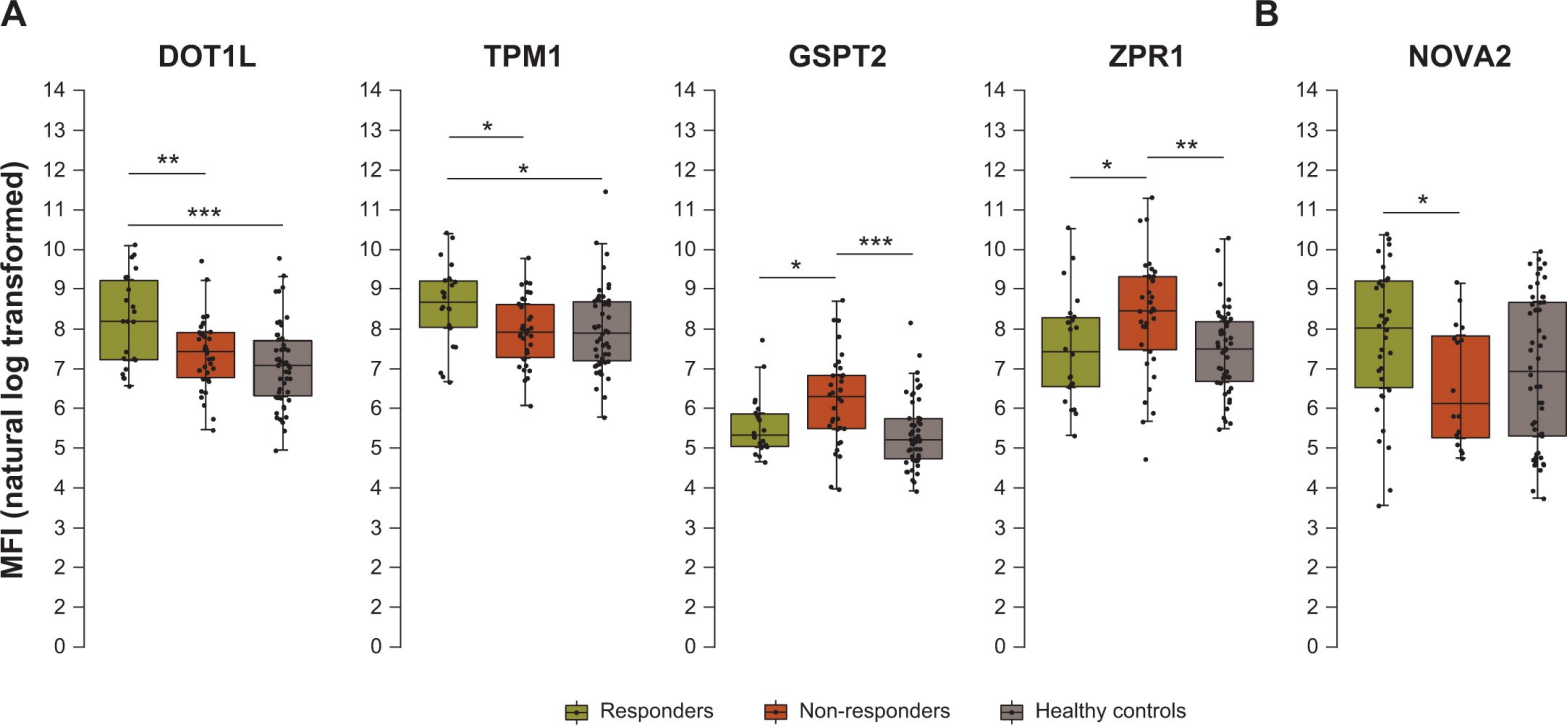
Tukey boxplots of the identified antigens in patients selected from the U-Act-Early. (**A**) methotrexate arm (n = 56) and (**B**) tocilizumab arm (n = 50) for responders and non-responders, plotted versus healthy controls. *p < 0.05, ** p < 0.01, ***p < 0.001. Whiskers display 1.5× interquartile range. Comparisons between groups were not corrected for multiple testing. DOT1L, DOT1-like histone lysine methyltransferase; GSPT2, G1 to S phase transition 2; MFI, median fluorescence intensity; NOVA2, neuro-oncological ventral antigen 2; TPM1, tropomyosin 1; ZPR1, zinc finger protein ZPR1.

In the methotrexate arm, the relevant antigens were DOT1-like histone lysine methyltransferase (DOT1L; coefficient 1.38 vs non-responder, p = 0.009), tropomyosin 1 (TPM1) (coefficient 1.70 vs non-responder, p = 0.003), G1 to S phase transition 2 (GSPT2) (coefficient –1.37 vs non-responder, p = 0.023) and the zinc finger protein ZPR1 (coefficient –1.17 vs non-responder, p = 0.021). In the tocilizumab arm, only neuro-oncological ventral antigen 2 (NOVA2) (coefficient 0.70 vs non-responder, p = 0.039) was relevant. When comparing the concentration of these antigens between non-responders and healthy controls, significant differences were found only in the methotrexate arm for GSPT2 (p < 0.001) and the zinc finger protein ZPR1 (p = 0.004), with lower values on average in the healthy controls, as expected (Fig 2). In the comparison between responders and matched healthy controls, significantly increased reactivity was noted for DOT1L (p < 0.001) and TPM1 (p = 0.033) in the responder group.

### Predictive accuracy

**Fig 3:**
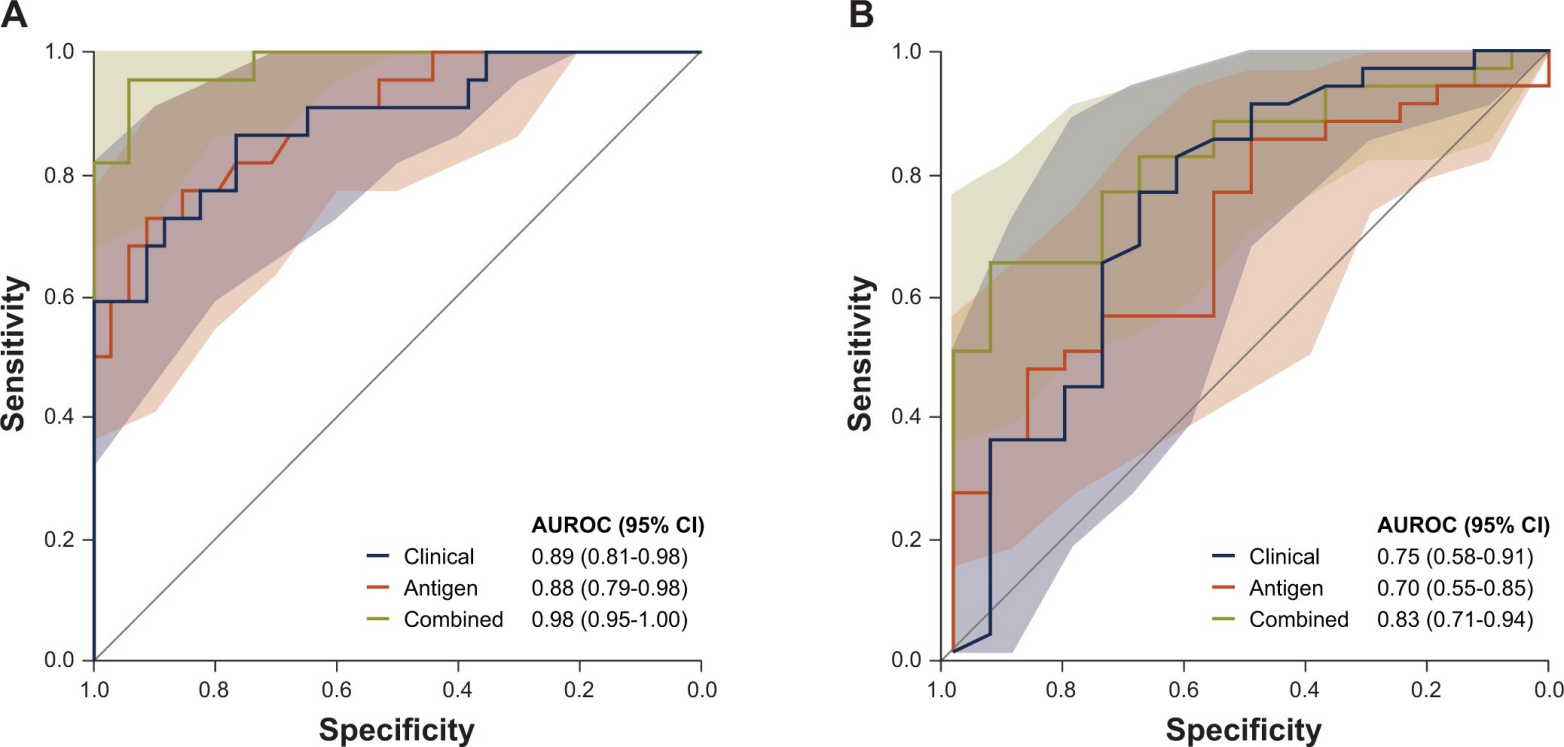
AUROC of the prediction models in patients selected from the U-Act-Early. (**A**) methotrexate^†^ arm and (**B**) tocilizumab^‡^ arm. ^†^The clinical model contains baseline DAS28 as predictor, and the antigen model contains baseline DOT1L, TPM1, GSPT2 and ZPR1 as predictors with group (responders vs non-responders) as outcome variables. The combined models contain both clinical and antigen predictors. ^‡^The clinical model contains baseline DAS28 as predictor, and the antigen model contains baseline NOVA2 as predictor with group (responders vs non-responders) as outcome variable. The combined models contain both clinical and antigen predictors. AUROC, area under the receiver operator characteristics curve with coloured areas indicating 95% CI of sensitivity; CI, confidence interval; DAS28, Disease Activity Score assessing 28 joints; DOT1L, DOT1-like histone lysine methyltransferase; GSPT2, G1 to S phase transition 2; MFI, median fluorescence intensity; NOVA2, neuro-oncological ventral antigen 2; TPM1, tropomyosin 1; ZPR1, zinc finger protein ZPR1.

In the univariable analyses of baseline clinical predictors, significant differences for responders versus non-responders in the methotrexate arm were observed only for DAS28 (p < 0.001) and in the tocilizumab arm only for body mass index (p = 0.049) and DAS28 (p = 0.007). After applying backward selection, DAS28 remained a significant clinical predictor (i.e. clinical model) in the tocilizumab arm. The discriminative accuracy of the clinical prediction model in the methotrexate arm was considered ‘good’ with an AUROC of 0.89 (95% CI, 0.81 to 0.98); in the tocilizumab arm it was considered ‘fair’ with an AUROC of 0.75 (95% CI, 0.59 to 0.91) (Fig 3).

The AUROC of the antigens (i.e. antigen model) in the methotrexate arm was 0.88 (95% CI, 0.79 to 0.98), indicating ‘good’ overall discriminative accuracy. When comparing the predictive accuracy of the antigen with that of the clinical model, no statistically significant difference (p = 0.88) was noted. However, the combined model (i.e. clinical plus antigen) showed significantly improved discrimination versus both the clinical (p = 0.040) and the antigen (p = 0.016) models. In the tocilizumab arm, the AUROC of the antigen model was 0.70 (95% CI, 0.55 to 0.85), corresponding with ‘fair’ accuracy; its discriminative accuracy was not statistically significantly lower (p ≤ 0.11) than either the combined model (AUROC, 0.82; 95% CI, 0.71 to 0.94) or the clinical model (AUROC, 0.74; 95%, CI 0.59 to 0.91).

### RF-positive or anti-CCP–positive RA

Additionally, we compared the concentrations of the antigens identified in the present analysis between seropositive, defined as RF-positive or anti-CCP–positive, and seronegative patients because these autoantibodies might be involved not only in the pathophysiology of RA but also in early treatment response, especially if that treatment response was to a methotrexate-based strategy [11]. In both the methotrexate and the tocilizumab arms, however, no statistically significant differences (p ≥ 0.06) were observed in antigen reactivities between these serological subgroups.

## Discussion

In baseline serum samples, we were able to identify autoantibodies to several antigens associated with increased or decreased reactivity in patients with DAS28 treatment response within the first 20 weeks of treat-to-target strategies. For this study we developed an antigen-coated, bead-based multiplex array composed of a comprehensive collection of antigens associated with rheumatic disease and RA [22]. Furthermore, we included a set of in vitro citrullinated proteins, which could be relevant to disease mechanisms such as cytokines, cytokine receptors, chemokines and proteinases. To evaluate whether these autoantibodies might be involved in pathways different from those in which RF and anti-CCP are involved, we compared the new autoantibody concentrations between patients with RF-positive and anti-CCP–positive and–negative disease and found no significant differences, prompting questioning of the pathological/aetiological role of autoantibodies in RA.

Antibodies to post-translationally modified proteins, such as ACPA and anti-carbamylated protein (anti-CarP), have a dominant role in the autoantibody spectrum in RA. In addition to these classical autoantibodies, anti-cytokine antibodies have been described in healthy subjects and patients with various autoimmune diseases, and they appear to exert immunomodulatory functions [27]. Interestingly, our analyses yielded only native predictive protein antigens, of which DOT1L is known to promote IL-6 and interferon-β production [28]. Recent studies have shown that DOT1L expression is increased in synovial tissue of RA patients and that it mediates the epigenetic regulation of chondrocyte and osteoclast differentiation [29]. Another identified antigen, NOVA2, is an alternative RNA-binding splicing regulator, expressed in adipose tissue [30] and in endothelial cells [31], and it plays a role in post-transcriptional regulation of vascular and neuronal functions. TPM1 encodes the tropomyosin α-1 chain, whereas anti-tropomyosin antibodies have been described in several rheumatic diseases such as Behçet's disease [32] and acute rheumatic fever [33]. The zinc finger protein ZPR1 binds to the cytoplasmic tyrosine kinase domain of inactive receptor tyrosine kinases and translocates to the nucleus in response to growth stimulatory signals, where it contributes to cell proliferation [34]. GSPT2 encodes the eukaryotic peptide chain release factor subunit 3b (eRF3) and is involved in protein biosynthesis and cytokinesis. eRF3 is an interacting partner of survivin, and both—together with anti-survivin antibodies—are prognostic biomarkers in RA, suggesting that the autoantibody response in RA might be directed against a protein complex of GSPT2 and survivin. Additionally, anti-GSPT2 antibodies were previously described as a diagnostic marker in early RA [22]. Whether these antigens have an aetiological role in RA remains to be investigated, but, based on our findings, they could help define a specific subgroup of patients who respond to methotrexate or tocilizumab induction therapy.

This study has a few limitations. Although there is a variety of antibody isotypes, each with different primary biological functions, we chose to measure only IgG antibodies because of their dominant role in humoural immunity. Nevertheless, other antibodies, particularly of the IgM type, could also be of interest in evaluating the treatment response of patients with early RA because their titres are elevated on antigen exposure before IgG antibody titres are elevated. In the current study, we focussed on profiling autoantibody reactivities to 45 well-known diagnostic rheumatic disease antigens and cytokines, cytokine receptors, chemokines and interferon family proteins to explore the autoantibody response to unmodified antigens. Other studies have investigated the antibody response to citrullinated peptide antigens compared with the anti-CCP2 assay in RA patients in more detail using high-density peptide arrays [35, 36]. Unfortunately, we did not measure other well-known autoantibodies in RA, such as anti-CCP2 [37] and anti-CarP [9], and therefore were unable to evaluate these concentrations and broaden the reliability of our assay. However, both approaches for testing unmodified and modified antigens could contribute to better understanding of the autoantibody reactivity profile of patients with early RA. Other apparent limitations of our study are the relatively small number of patients included in the analyses and the absence of a validation cohort. To address this, we used a supervised clustering method to detect candidate autoantibodies, suited for handling both collinearity and data sets consisting of more observations than samples [38]. In addition, in the present analyses, patients who were neither responders nor non-responders were not included, which might explain the high predictive accuracy found for the antigens. This could also have impaired the reproducibility of our prediction model in other patient cohorts. Furthermore, because sera in U-Act-Early were collected cross-sectionally at baseline before patients received their first dose of medication, we were not able to determine whether titres of the identified antibodies changed in responders after treatment. Therefore, further research is required to evaluate how these biomarker concentrations correlate with disease activity over time. To date, no infallible diagnostic test is available for RA. Although all patients who participated in the U-Act-Early study fulfilled the 1987 American College of Rheumatology (ACR) or 2010 ACR/European League Against Rheumatism classification criteria, which take into account the likelihood of chronic polyarthritis developing [18], these criteria have limited diagnostic accuracy on an individual level [1]. Considering the exceptionally short symptom duration of the patients included in the U-Act-Early study and, thus, in the present analysis, it was not proven that chronic RA would develop in all patients. The strength of the present study is that, to the best of our knowledge, it is the first to investigate on this scale a broad panel of IgG antibodies in patients with early RA before they began their first DMARD therapy. Although patients in U-Act-Early were randomly allocated and demonstrated similar clinical characteristics at baseline, within the first 20 weeks a clearly different pattern of disease activity was noted between patients (responders vs non-responders) in both treatment arms selected in the present analysis, which potentially could indicate a varying underlying pathophysiology in this relatively homogeneous study population. This finding might further underscore the necessity of applying personalised medicine, guided by prognostic (bio)markers, to better select optimal therapeutic strategies and further improve treatment outcomes in patients with early RA.

## Conclusions

In summary, comprehensive profiling of novel antigens revealed several prognostic candidate IgG autoantibodies with promising predictive accuracy for early treatment response in patients with newly diagnosed RA. Further research, however, is required to establish the predictive accuracy of these markers among different populations before they can be applied in clinical practice. Given that different predictive autoantibodies—each targeting a specific pathway—were identified for each treatment arm, it appears that the likelihood for a good response to therapy is dependent on both the concentration of IgG autoantibodies at baseline and the type of treatment subsequently initiated.

## Supporting information

S1 File Overview of measured antigens. (XLSX)

S2 File Anonymised data set. (XLSX)

S1 Table Overview of measured antigens. (DOCX)

S1 Data (XLSX)
