# Supplementary material for: Comprehensive exploratory autoantibody profiling in patients with early rheumatoid arthritis treated with methotrexate or tocilizumab
Source: PLoS One. 2020 Dec 10;15(12):e0241189. doi: 10.1371/journal.pone.0241189 (PMC7728181; doi:10.1371/journal.pone.0241189)
Supplement: S1 Table — (DOCX) [file pone.0241189.s003.docx]

| **Supplementary file 1** Overview of measured antigens | | | |
| --- | --- | --- | --- |
| **Gene ID** | **Gene symbol** | **Gene name** | **Type** |
| 348 | APOE | apolipoprotein E | Citrullinated |
| 5937 | RBMS1 | RNA binding motif, single stranded interacting protein 1 | Citrullinated |
| 633 | BGN | biglycan | Citrullinated |
| 2243 | FGA | fibrinogen alpha chain | Citrullinated |
| 6374 | CXCL5 | chemokine (C-X-C motif) ligand 5 | Citrullinated |
| 3178 | HNRNPA1 | heterogeneous nuclear ribonucleoprotein A1 | Citrullinated |
| 7431 | VIM | vimentin | Citrullinated |
| 165 | AEBP1 | AE binding protein 1 | Citrullinated |
| 6434 | TRA2B | transformer 2 beta homolog (Drosophila) | Citrullinated |
| 3017 | HIST1H2BD | histone cluster 1, H2bd | Citrullinated |
| 8320 | EOMES | eomesodermin | Citrullinated |
| 2335 | FN1 | fibronectin 1 | Citrullinated |
| 811 | CALR | calreticulin | Citrullinated |
| 2023 | ENO1 | enolase 1, (alpha) | Citrullinated |
| 3329 | HSPD1 | heat shock 60kDa protein 1 (chaperonin) | Citrullinated |
| 8359 | HIST1H4A | histone cluster 1, H4a | Citrullinated |
| 23569 | PADI4 | peptidyl arginine deiminase, type IV | Citrullinated |
| 5725 | PTBP1 | polypyrimidine tract binding protein 1 | Citrullinated |
| 2237 | FEN1 | flap structure-specific endonuclease 1 | Citrullinated |
| 1655 | DDX5 | DEAD (Asp-Glu-Ala-Asp) box helicase 5 | Citrullinated |
| 6421 | SFPQ | splicing factor proline/glutamine-rich | Citrullinated |
| 203068 | TUBB | tubulin, beta class I | Citrullinated |
| 4001 | LMNB1 | lamin B1 | Citrullinated |
| 4869 | NPM1 | nucleophosmin (nucleolar phosphoprotein B23, numatrin) | Citrullinated |
| 9188 | DDX21 | DEAD (Asp-Glu-Ala-Asp) box helicase 21 | Citrullinated |
| 2244 | FGB | fibrinogen beta chain | Citrullinated |
| 8623 | ASMTL | acetylserotonin O-methyltransferase-like | Citrullinated |
| 6696 | SPP1 | secreted phosphoprotein 1 | Citrullinated |
| 10130 | PDIA6 | protein disulfide isomerase family A, member 6 | Citrullinated |
| 3371 | TNC | tenascin C | Citrullinated |
| 60 | ACTB | actin, beta | Citrullinated |
| 3479 | IGF1 | insulin-like growth factor 1 (somatomedin C) | Citrullinated |
| 11052 | CPSF6 | cleavage and polyadenylation specific factor 6, 68kDa | Citrullinated |
| 4171 | MCM2 | minichromosome maintenance complex component 2 | Citrullinated |
| 3337 | DNAJB1 | DnaJ (Hsp40) homolog, subfamily B, member 1 | Citrullinated |
| 1191 | CLU | clusterin | Citrullinated |
| 9584 | RBM39 | RNA binding motif protein 39 | Citrullinated |
| 3489 | IGFBP6 | insulin-like growth factor binding protein 6 | Citrullinated |
| 4841 | NONO | non-POU domain containing, octamer-binding | Citrullinated |
| 6432 | SRSF7 | serine/arginine-rich splicing factor 7 | Citrullinated |
| 7458 | EIF4H | eukaryotic translation initiation factor 4H | Citrullinated |
| 115650 | TNFRSF13C | TNF receptor superfamily member 13C | Noncitrullinated |
| 8742 | TNFSF12 | tumor necrosis factor superfamily member 12 | Noncitrullinated |
| 933 | CD22 | CD22 molecule | Noncitrullinated |
| 8741 | TNFSF13 | tumor necrosis factor superfamily member 13 | Noncitrullinated |
| 6352 | CCL5 | C-C motif chemokine ligand 5 | Noncitrullinated |
| 941 | CD80 | CD80 molecule | Noncitrullinated |
| 1438 | CSF2RA | colony stimulating factor 2 receptor alpha subunit | Noncitrullinated |
| 348 | APOE | apolipoprotein E | Noncitrullinated |
| 5937 | RBMS1 | RNA binding motif, single stranded interacting protein 1 | Noncitrullinated |
| 2243 | FGA | fibrinogen alpha chain | Noncitrullinated |
| 6374 | CXCL5 | chemokine (C-X-C motif) ligand 5 | Noncitrullinated |
| 3178 | HNRNPA1 | heterogeneous nuclear ribonucleoprotein A1 | Noncitrullinated |
| 7431 | VIM | vimentin | Noncitrullinated |

| **Supplementary file 1** Overview of measured antigens (*continued*) | | | |
| --- | --- | --- | --- |
| **Gene ID** | **Gene symbol** | **Gene name** | **Type** |
| 165 | AEBP1 | AE binding protein 1 | Noncitrullinated |
| 6434 | TRA2B | transformer 2 beta homolog (Drosophila) | Noncitrullinated |
| 3017 | HIST1H2BD | histone cluster 1, H2bd | Noncitrullinated |
| 8320 | EOMES | eomesodermin | Noncitrullinated |
| 2335 | FN1 | fibronectin 1 | Noncitrullinated |
| 811 | CALR | calreticulin | Noncitrullinated |
| 2023 | ENO1 | enolase 1, (alpha) | Noncitrullinated |
| 3329 | HSPD1 | heat shock 60kDa protein 1 (chaperonin) | Noncitrullinated |
| 8359 | HIST1H4A | histone cluster 1, H4a | Noncitrullinated |
| 23569 | PADI4 | peptidyl arginine deiminase, type IV | Noncitrullinated |
| 5725 | PTBP1 | polypyrimidine tract binding protein 1 | Noncitrullinated |
| 2237 | FEN1 | flap structure-specific endonuclease 1 | Noncitrullinated |
| 1655 | DDX5 | DEAD (Asp-Glu-Ala-Asp) box helicase 5 | Noncitrullinated |
| 6421 | SFPQ | splicing factor proline/glutamine-rich | Noncitrullinated |
| 203068 | TUBB | tubulin, beta class I | Noncitrullinated |
| 4001 | LMNB1 | lamin B1 | Noncitrullinated |
| 4869 | NPM1 | nucleophosmin (nucleolar phosphoprotein B23, numatrin) | Noncitrullinated |
| 9188 | DDX21 | DEAD (Asp-Glu-Ala-Asp) box helicase 21 | Noncitrullinated |
| 2244 | FGB | fibrinogen beta chain | Noncitrullinated |
| 8623 | ASMTL | acetylserotonin O-methyltransferase-like | Noncitrullinated |
| 6696 | SPP1 | secreted phosphoprotein 1 | Noncitrullinated |
| 10130 | PDIA6 | protein disulfide isomerase family A, member 6 | Noncitrullinated |
| 3371 | TNC | tenascin C | Noncitrullinated |
| 60 | ACTB | actin, beta | Noncitrullinated |
| 3479 | IGF1 | insulin-like growth factor 1 (somatomedin C) | Noncitrullinated |
| 11052 | CPSF6 | cleavage and polyadenylation specific factor 6, 68kDa | Noncitrullinated |
| 4171 | MCM2 | minichromosome maintenance complex component 2 | Noncitrullinated |
| 3337 | DNAJB1 | DnaJ (Hsp40) homolog, subfamily B, member 1 | Noncitrullinated |
| 1191 | CLU | clusterin | Noncitrullinated |
| 9584 | RBM39 | RNA binding motif protein 39 | Noncitrullinated |
| 3489 | IGFBP6 | insulin-like growth factor binding protein 6 | Noncitrullinated |
| 4841 | NONO | non-POU domain containing, octamer-binding | Noncitrullinated |
| 6432 | SRSF7 | serine/arginine-rich splicing factor 7 | Noncitrullinated |
| 7458 | EIF4H | eukaryotic translation initiation factor 4H | Noncitrullinated |
| 3570 | IL6R | interleukin 6 receptor | Noncitrullinated |
| 940 | CD28 | CD28 molecule | Noncitrullinated |
| 3440 | IFNA2 | interferon alpha 2 | Noncitrullinated |
| 3718 | JAK3 | Janus kinase 3 | Noncitrullinated |
| 1639 | DCTN1 | dynactin subunit 1 | Noncitrullinated |
| 942 | CD86 | CD86 molecule | Noncitrullinated |
| 3454 | IFNAR1 | Interferon-alpha/beta Receptor 1 | Noncitrullinated |
| 10673 | TNFSF13B | tumor necrosis factor superfamily member 13b | Noncitrullinated |
| 6347 | CCL2 | C-C motif chemokine ligand 2 | Noncitrullinated |
| 920 | CD4 | CD4 molecule | Noncitrullinated |
| 23765 | IL17RA | interleukin 17 receptor A | Noncitrullinated |
| 3553 | IL1B | interleukin 1, beta | Noncitrullinated |
| 9988 | DMTF1 | cyclin D binding myb-like transcription factor 1 | Noncitrullinated |
| 1639 | DCTN1 | dynactin 1 | Noncitrullinated |
| 10970 | CKAP4 | cytoskeleton-associated protein 4 | Noncitrullinated |
| 3561 | IL2RG | interleukin 2 receptor, gamma | Noncitrullinated |
| 712 | C1QA | complement component 1, q subcomponent, A chain | Noncitrullinated |
| 931 | MS4A1 | membrane-spanning 4-domains, subfamily A, member 1 | Noncitrullinated |
| 1108 | CHD4 | chromodomain helicase DNA binding protein 4 | Noncitrullinated |
| 8079 | MLF2 | myeloid leukemia factor 2 | Noncitrullinated |
| 6366 | CCL21 | chemokine (C-C motif) ligand 21 | Noncitrullinated |
| 7423 | VEGFB | vascular endothelial growth factor B | Noncitrullinated |
| 6279 | S100A8 | S100 calcium binding protein A8 | Noncitrullinated |
| 3552 | IL1A | interleukin 1, alpha | Noncitrullinated |

| **Supplementary file 1** Overview of measured antigens (*continued*) | | | |
| --- | --- | --- | --- |
| **Gene ID** | **Gene symbol** | **Gene name** | **Type** |
| 4314 | MMP3 | matrix metallopeptidase 3 (stromelysin 1, progelatinase) | Noncitrullinated |
| 3600 | IL15 | interleukin 15 | Noncitrullinated |
| 7124 | TNF | tumor necrosis factor | Noncitrullinated |
| 211 | ALAS1 | 5'-aminolevulinate synthase 1 | Noncitrullinated |
| 204 | AK2 | adenylate kinase 2 | Noncitrullinated |
| 23301 | EHBP1 | EH domain binding protein 1 | Noncitrullinated |
| 112950 | MED8 | mediator complex subunit 8 | Noncitrullinated |
| 10528 | NOP56 | NOP56 ribonucleoprotein | Noncitrullinated |
| 10969 | EBNA1BP2 | EBNA1 binding protein 2 | Noncitrullinated |
| 54521 | WDR44 | WD repeat domain 44 | Noncitrullinated |
| 65109 | UPF3B | UPF3 regulator of nonsense transcripts homolog B (yeast) | Noncitrullinated |
| 23256 | SCFD1 | sec1 family domain containing 1 | Noncitrullinated |
| 8882 | ZPR1 | ZPR1 zinc finger | Noncitrullinated |
| 9727 | RAB11FIP3 | RAB11 family interacting protein 3 (class II) | Noncitrullinated |
| 128061 | C1orf131 | chromosome 1 open reading frame 131 | Noncitrullinated |
| 6091 | ROBO1 | roundabout, axon guidance receptor, homolog 1 (Drosophila) | Noncitrullinated |
| 90416 | C15orf57 | chromosome 15 open reading frame 57 | Noncitrullinated |
| 128061 | C1orf131 | chromosome 1 open reading frame 131 | Noncitrullinated |
| 30827 | CXXC1 | CXXC finger protein 1 | Noncitrullinated |
| 11093 | ADAMTS13 | ADAM metallopeptidase with thrombospondin type 1 motif, 13 | Noncitrullinated |
| 65003 | MRPL11 | mitochondrial ribosomal protein L11 | Noncitrullinated |
| 8795 | TNFRSF10B | tumor necrosis factor receptor superfamily, member 10b | Noncitrullinated |
| 10290 | SPEG | SPEG complex locus | Noncitrullinated |
| 2934 | GSN | gelsolin | Noncitrullinated |
| 9315 | NREP | neuronal regeneration related protein | Noncitrullinated |
| 4357 | MPST | mercaptopyruvate sulfurtransferase | Noncitrullinated |
| 84687 | PPP1R9B | protein phosphatase 1, regulatory subunit 9B | Noncitrullinated |
| 30 | ACAA1 | acetyl-CoA acyltransferase 1 | Noncitrullinated |
| 51561 | IL23A | interleukin 23, alpha subunit p19 | Noncitrullinated |
| 282617 | IFNL3 | interferon, lambda 3 | Noncitrullinated |
| 6355 | CCL8 | chemokine (C-C motif) ligand 8 | Noncitrullinated |
| 55801 | IL26 | interleukin 26 | Noncitrullinated |
| 84639 | IL1F10 | interleukin 1 family, member 10 (theta) | Noncitrullinated |
| 3576 | CXCL8 | chemokine (C-X-C motif) ligand 8 | Noncitrullinated |
| 282616 | IFNL2 | interferon, lambda 2 | Noncitrullinated |
| 26525 | IL36RN | interleukin 36 receptor antagonist | Noncitrullinated |
| 6358 | CCL14 | chemokine (C-C motif) ligand 14 | Noncitrullinated |
| 64806 | IL25 | interleukin 25 | Noncitrullinated |
| 3606 | IL18 | interleukin 18 | Noncitrullinated |
| 27178 | IL37 | interleukin 37 | Noncitrullinated |
| 50616 | IL22 | interleukin 22 | Noncitrullinated |
| 59067 | IL21 | interleukin 21 | Noncitrullinated |
| 246778 | IL27 | interleukin 27 | Noncitrullinated |
| 27179 | IL36A | interleukin 36, alpha | Noncitrullinated |
| 6354 | CCL7 | chemokine (C-C motif) ligand 7 | Noncitrullinated |
| 3605 | IL17A | interleukin 17A | Noncitrullinated |
| 56300 | IL36G | interleukin 36, gamma | Noncitrullinated |
| 6370 | CCL25 | chemokine (C-C motif) ligand 25 | Noncitrullinated |
| 6356 | CCL11 | chemokine (C-C motif) ligand 11 | Noncitrullinated |
| 90865 | IL33 | interleukin 33 | Noncitrullinated |
| 39 | ACAT2 | acetyl-CoA acetyltransferase 2 | Noncitrullinated |
| 8473 | OGT | O-linked N-acetylglucosamine (GlcNAc) transferase | Noncitrullinated |
| 57498 | KIDINS220 | kinase D-interacting substrate, 220kDa | Noncitrullinated |
| 10961 | ERP29 | endoplasmic reticulum protein 29 | Noncitrullinated |
| 91544 | UBXN11 | UBX domain protein 11 | Noncitrullinated |
| 3798 | KIF5A | kinesin family member 5A | Noncitrullinated |
| 3182 | HNRNPAB | heterogeneous nuclear ribonucleoprotein A/B | Noncitrullinated |
| 59307 | SIGIRR | single immunoglobulin and toll-interleukin 1 receptor (TIR) domain | Noncitrullinated |

| **Supplementary file 1** Overview of measured antigens (*continued*) | | | |
| --- | --- | --- | --- |
| **Gene ID** | **Gene symbol** | **Gene name** | **Type** |
| 9704 | DHX34 | DEAH (Asp-Glu-Ala-His) box polypeptide 34 | Noncitrullinated |
| 25855 | BRMS1 | breast cancer metastasis suppressor 1 | Noncitrullinated |
| 5589 | PRKCSH | protein kinase C substrate 80K-H | Noncitrullinated |
| 6234 | RPS28 | ribosomal protein S28 | Noncitrullinated |
| 11075 | STMN2 | stathmin 2 | Noncitrullinated |
| 4043 | LRPAP1 | low density lipoprotein receptor-related protein associated protein 1 | Noncitrullinated |
| 84444 | DOT1L | DOT1-like histone lysine methyltransferase | Noncitrullinated |
| 10483 | SEC23B | Sec23 homolog B (S. cerevisiae) | Noncitrullinated |
| 6449 | SGTA | small glutamine-rich tetratricopeptide repeat (TPR)-containing, alpha | Noncitrullinated |
| 26515 | TIMM10B | translocase of inner mitochondrial membrane 10 homolog B (yeast) | Noncitrullinated |
| 64762 | GAREM | GRB2 associated, regulator of MAPK1 | Noncitrullinated |
| 10422 | UBAC1 | UBA domain containing 1 | Noncitrullinated |
| 89891 | WDR34 | WD repeat domain 34 | Noncitrullinated |
| 90525 | SHF | Src homology 2 domain containing F | Noncitrullinated |
| 6240 | RRM1 | ribonucleotide reductase M1 | Noncitrullinated |
| 5494 | PPM1A | protein phosphatase, Mg2+/Mn2+ dependent, 1A | Noncitrullinated |
| 23002 | DAAM1 | dishevelled associated activator of morphogenesis 1 | Noncitrullinated |
| 79714 | CCDC51 | coiled-coil domain containing 51 | Noncitrullinated |
| 1485 | CTAG1B | cancer/testis antigen 1B | Noncitrullinated |
| 22937 | SCAP | SREBF chaperone | Noncitrullinated |
| 5834 | PYGB | phosphorylase, glycogen; brain | Noncitrullinated |
| 64062 | RBM26 | RNA binding motif protein 26 | Noncitrullinated |
| 3059 | HCLS1 | hematopoietic cell-specific Lyn substrate 1 | Noncitrullinated |
| 84064 | HDHD2 | haloacid dehalogenase-like hydrolase domain containing 2 | Noncitrullinated |
| 29094 | LGALSL | lectin, galactoside-binding-like | Noncitrullinated |
| 9360 | PPIG | peptidylprolyl isomerase G (cyclophilin G) | Noncitrullinated |
| 6249 | CLIP1 | CAP-GLY domain containing linker protein 1 | Noncitrullinated |
| 79668 | PARP8 | poly (ADP-ribose) polymerase family, member 8 | Noncitrullinated |
| 23762 | OSBP2 | oxysterol binding protein 2 | Noncitrullinated |
| 55118 | CRTAC1 | cartilage acidic protein 1 | Noncitrullinated |
| 55850 | USE1 | unconventional SNARE in the ER 1 homolog (S. cerevisiae) | Noncitrullinated |
| 3281 | HSBP1 | heat shock factor binding protein 1 | Noncitrullinated |
| 57572 | DOCK6 | dedicator of cytokinesis 6 | Noncitrullinated |
| 8608 | RDH16 | retinol dehydrogenase 16 (all-trans) | Noncitrullinated |
| 58516 | FAM60A | family with sequence similarity 60, member A | Noncitrullinated |
| 5187 | PER1 | period circadian clock 1 | Noncitrullinated |
| 26275 | HIBCH | 3-hydroxyisobutyryl-CoA hydrolase | Noncitrullinated |
| 5174 | PDZK1 | PDZ domain containing 1 | Noncitrullinated |
| 117584 | RFFL | ring finger and FYVE-like domain containing E3 ubiquitin protein ligase | Noncitrullinated |
| 11170 | FAM107A | family with sequence similarity 107, member A | Noncitrullinated |
| 23479 | ISCU | iron-sulphur cluster assembly enzyme | Noncitrullinated |
| 7185 | TRAF1 | TNF receptor-associated factor 1 | Noncitrullinated |
| 10476 | ATP5H | ATP synthase, H+ transporting, mitochondrial Fo complex, subunit d | Noncitrullinated |
| 23645 | PPP1R15A | protein phosphatase 1, regulatory subunit 15A | Noncitrullinated |
| 83986 | ITFG3 | integrin alpha FG-GAP repeat containing 3 | Noncitrullinated |
| 54856 | GON4L | gon-4-like (C. elegans) | Noncitrullinated |
| 3875 | KRT18 | keratin 18, type I | Noncitrullinated |
| 158471 | PRUNE2 | prune homolog 2 (Drosophila) | Noncitrullinated |
| 2922 | GRP | gastrin-releasing peptide | Noncitrullinated |
| 9909 | DENND4B | DENN/MADD domain containing 4B | Noncitrullinated |
| 4313 | MMP2 | matrix metallopeptidase 2 | Noncitrullinated |
| 6280 | S100A9 | S100 calcium binding protein A9 | Noncitrullinated |
| 4282 | MIF | macrophage migration inhibitory factor (glycosylation-inhibiting factor) | Noncitrullinated |
| 91012 | CERS5 | ceramide synthase 5 | Noncitrullinated |
| 80781 | COL18A1 | collagen, type XVIII, alpha 1 | Noncitrullinated |
| 7525 | YES1 | YES proto-oncogene 1, Src family tyrosine kinase | Noncitrullinated |
| 3855 | KRT7 | keratin 7, type II | Noncitrullinated |
| 4316 | MMP7 | matrix metallopeptidase 7 (matrilysin, uterine) | Noncitrullinated |

| **Supplementary file 1** Overview of measured antigens (*continued*) | | | |
| --- | --- | --- | --- |
| **GeneID** | **Gene symbol** | **Gene name** | **Type** |
| 118430 | MUCL1 | mucin-like 1 | Noncitrullinated |
| 55131 | RBM28 | RNA binding motif protein 28 | Noncitrullinated |
| 51599 | LSR | lipolysis stimulated lipoprotein receptor | Noncitrullinated |
| 56950 | SMYD2 | SET and MYND domain containing 2 | Noncitrullinated |
| 8740 | TNFSF14 | tumour necrosis factor (ligand) superfamily, member 14 | Noncitrullinated |
| 26291 | FGF21 | fibroblast growth factor 21 | Noncitrullinated |
| 6197 | RPS6KA3 | ribosomal protein S6 kinase, 90kDa, polypeptide 3 | Noncitrullinated |
| 54853 | WDR55 | WD repeat domain 55 | Noncitrullinated |
| 9112 | MTA1 | metastasis associated 1 | Noncitrullinated |
| 5716 | PSMD10 | proteasome (prosome, macropain) 26S subunit, non-ATPase, 10 | Noncitrullinated |
| 4150 | MAZ | MYC-associated zinc finger protein (purine-binding transcription factor) | Noncitrullinated |
| 7168 | TPM1 | tropomyosin 1 (alpha) | Noncitrullinated |
| 5764 | PTN | pleiotrophin | Noncitrullinated |
| 6136 | RPL12 | ribosomal protein L12 | Noncitrullinated |
| 3818 | KLKB1 | kallikrein B, plasma (Fletcher factor) 1 | Noncitrullinated |
| 3880 | KRT19 | keratin 19, type I | Noncitrullinated |
| 10916 | MAGED2 | melanoma antigen family D, 2 | Noncitrullinated |
| 1281 | COL3A1 | collagen, type III, alpha 1 | Noncitrullinated |
| 26146 | TRAF3IP1 | TNF receptor-associated factor 3 interacting protein 1 | Noncitrullinated |
| 23646 | PLD3 | phospholipase D family, member 3 | Noncitrullinated |
| 3439 | IFNA1 | interferon, alpha 1 | Noncitrullinated |
| 4676 | NAP1L4 | nucleosome assembly protein 1-like 4 | Noncitrullinated |
| 90557 | CCDC74A | coiled-coil domain containing 74A | Noncitrullinated |
| 51011 | FAHD2A, | fumarylacetoacetate hydrolase domain containing 2A, | Noncitrullinated |
| 151313 | FAHD2B | fumarylacetoacetate hydrolase domain containing 2B | Noncitrullinated |
| 2919 | CXCL1 | chemokine (C-X-C motif) ligand 1 | Noncitrullinated |
| 1508 | CTSB | cathepsin B | Noncitrullinated |
| 9531 | BAG3 | BCL2-associated athanogene 3 | Noncitrullinated |
| 834 | CASP1 | caspase 1, apoptosis-related cysteine peptidase | Noncitrullinated |
| 840 | CASP7 | caspase 7, apoptosis-related cysteine peptidase | Noncitrullinated |
| 4322 | MMP13 | matrix metallopeptidase 13 (collagenase 3) | Noncitrullinated |
| 64135 | IFIH1 | interferon induced with helicase C domain 1 | Noncitrullinated |
| 843 | CASP10 | caspase 10, apoptosis-related cysteine peptidase | Noncitrullinated |
| 3856 | KRT8 | keratin 8, type II | Noncitrullinated |
| 523 | ATP6V1A | ATPase, H+ transporting, lysosomal 70kDa, V1 subunit A | Noncitrullinated |
| 130617 | ZFAND2B | zinc finger, AN1-type domain 2B | Noncitrullinated |
| 4000 | LMNA | lamin A/C | Noncitrullinated |
| 55830 | GLT8D1 | glycosyltransferase 8 domain containing 1 | Noncitrullinated |
| 3559 | IL2RA | interleukin 2 receptor, alpha | Noncitrullinated |
| 54474 | KRT20 | keratin 20, type I | Noncitrullinated |
| 2081 | ERN1 | endoplasmic reticulum to nucleus signaling 1 | Noncitrullinated |
| 3593 | IL12B | interleukin 12B | Noncitrullinated |
| 10491 | CRTAP | cartilage associated protein | Noncitrullinated |
| 721 | C4B | complement component 4B (Chido blood group) | Noncitrullinated |
| 23647 | ARFIP2 | ADP-ribosylation factor interacting protein 2 | Noncitrullinated |
| 1287 | COL4A5 | collagen, type IV, alpha 5 | Noncitrullinated |
| 9173 | IL1RL1 | interleukin 1 receptor-like 1 | Noncitrullinated |
| 733 | C8G | complement component 8, gamma polypeptide | Noncitrullinated |
| 2921 | CXCL3 | chemokine (C-X-C motif) ligand 3 | Noncitrullinated |
| 1665 | DHX15 | DEAH (Asp-Glu-Ala-His) box helicase 15 | Noncitrullinated |
| 90736 | FAM104B | family with sequence similarity 104, member B | Noncitrullinated |
| 66008 | TRAK2 | trafficking protein, kinesin binding 2 | Noncitrullinated |
| 10561 | IFI44 | interferon-induced protein 44 | Noncitrullinated |
| 3443 | IFNA6 | interferon, alpha 6 | Noncitrullinated |
| 3460 | IFNGR2 | interferon gamma receptor 2 (interferon gamma transducer 1) | Noncitrullinated |
| 4326 | MMP17 | matrix metallopeptidase 17 (membrane-inserted) | Noncitrullinated |
| 713 | C1QB | complement component 1, q subcomponent, B chain | Noncitrullinated |
| 7133 | TNFRSF1B | tumour necrosis factor receptor superfamily, member 1B | Noncitrullinated |

| **Supplementary file 1** Overview of measured antigens (*continued*) | | | |
| --- | --- | --- | --- |
| **Gene ID** | **Gene symbol** | **Gene name** | **Type** |
| 720 | C4A | complement component 4A (Rodgers blood group) | Noncitrullinated |
| 3437 | IFIT3 | interferon-induced protein with tetratricopeptide repeats 3 | Noncitrullinated |
| 629 | CFB | complement factor B | Noncitrullinated |
| 3446 | IFNA10 | interferon, alpha 10 | Noncitrullinated |
| 732 | C8B | complement component 8, beta polypeptide | Noncitrullinated |
| 3441 | IFNA4 | interferon, alpha 4 | Noncitrullinated |
| 84572 | GNPTG | N-acetylglucosamine-1-phosphate transferase, gamma subunit | Noncitrullinated |
| 3934 | LCN2 | lipocalin 2 | Noncitrullinated |
| 10878 | CFHR3 | complement factor H-related 3 | Noncitrullinated |
| 93145 | OLFM2 | olfactomedin 2 | Noncitrullinated |
| 1509 | CTSD | cathepsin D | Noncitrullinated |
| 7076 | TIMP1 | TIMP metallopeptidase inhibitor 1 | Noncitrullinated |
| 56970 | ATXN7L3 | ataxin 7-like 3 | Noncitrullinated |
| 1181 | CLCN2 | chloride channel, voltage-sensitive 2 | Noncitrullinated |
| 66005 | CHID1 | chitinase domain containing 1 | Noncitrullinated |
| 7186 | TRAF2 | TNF receptor-associated factor 2 | Noncitrullinated |
| 150946 | GAREML | GRB2 associated, regulator of MAPK1-like | Noncitrullinated |
| 1514 | CTSL | cathepsin L | Noncitrullinated |
| 7097 | TLR2 | toll-like receptor 2 | Noncitrullinated |
| 10015 | PDCD6IP | programmed cell death 6 interacting protein | Noncitrullinated |
| 118471 | PRAP1 | proline-rich acidic protein 1 | Noncitrullinated |
| 23363 | OBSL1 | obscurin-like 1 | Noncitrullinated |
| 7174 | TPP2 | tripeptidyl peptidase II | Noncitrullinated |
| 1521 | CTSW | cathepsin W | Noncitrullinated |
| 3697 | ITIH1 | inter-alpha-trypsin inhibitor heavy chain 1 | Noncitrullinated |
| 1284 | COL4A2 | collagen, type IV, alpha 2 | Noncitrullinated |
| 26578 | OSTF1 | osteoclast stimulating factor 1 | Noncitrullinated |
| 7126 | TNFAIP1 | tumour necrosis factor, alpha-induced protein 1 (endothelial) | Noncitrullinated |
| 1512 | CTSH | cathepsin H | Noncitrullinated |
| 131544 | CRYBG3 | beta-gamma crystallin domain containing 3 | Noncitrullinated |
| 2920 | CXCL2 | chemokine (C-X-C motif) ligand 2 | Noncitrullinated |
| 835 | CASP2 | caspase 2, apoptosis-related cysteine peptidase | Noncitrullinated |
| 7128 | TNFAIP3 | tumour necrosis factor, alpha-induced protein 3 | Noncitrullinated |
| 3557 | IL1RN | interleukin 1 receptor antagonist | Noncitrullinated |
| 1116 | CHI3L1 | chitinase 3-like 1 (cartilage glycoprotein-39) | Noncitrullinated |
| 3603 | IL16 | interleukin 16 | Noncitrullinated |
| 3554 | IL1R1 | interleukin 1 receptor, type I | Noncitrullinated |
| 3659 | IRF1 | interferon regulatory factor 1 | Noncitrullinated |
| 55421 | C17orf85 | chromosome 17 open reading frame 85 | Noncitrullinated |
| 3572 | IL6ST | interleukin 6 signal transducer | Noncitrullinated |
| 3562 | IL3 | interleukin 3 | Noncitrullinated |
| 51135 | IRAK4 | interleukin-1 receptor-associated kinase 4 | Noncitrullinated |
| 3902 | LAG3 | lymphocyte-activation gene 3 | Noncitrullinated |
| 5155 | PDGFB | platelet-derived growth factor beta polypeptide | Noncitrullinated |
| 3586 | IL10 | interleukin 10 | Noncitrullinated |
| 3566 | IL4R | interleukin 4 receptor | Noncitrullinated |
| 6351 | CCL4 | chemokine (C-C motif) ligand 4 | Noncitrullinated |
| 5154 | PDGFA | platelet-derived growth factor alpha polypeptide | Noncitrullinated |
| 3662 | IRF4 | interferon regulatory factor 4 | Noncitrullinated |
| 79148 | MMP28 | matrix metallopeptidase 28 | Noncitrullinated |
| 1960 | EGR3 | early growth response 3 | Noncitrullinated |
| 9894 | TELO2 | telomere maintenance 2 | Noncitrullinated |
| 11019 | LIAS | lipoic acid synthetase | Noncitrullinated |
| 841 | CASP8 | caspase 8, apoptosis-related cysteine peptidase | Noncitrullinated |
| 3854 | KRT6B | keratin 6B, type II | Noncitrullinated |
| 838 | CASP5 | caspase 5, apoptosis-related cysteine peptidase | Noncitrullinated |
| 3853 | KRT6A | keratin 6A, type II | Noncitrullinated |
| 1515 | CTSV | cathepsin V | Noncitrullinated |

| **Supplementary file 1** Overview of measured antigens (*continued*) | | | |
| --- | --- | --- | --- |
| **Gene ID** | **Gene symbol** | **Gene name** | **Type** |
| 4321 | MMP12 | matrix metallopeptidase 12 (macrophage elastase) | Noncitrullinated |
| 57062 | DDX24 | DEAD (Asp-Glu-Ala-Asp) box helicase 24 | Noncitrullinated |
| 27344 | PCSK1N | proprotein convertase subtilisin/kexin type 1 inhibitor | Noncitrullinated |
| 84893 | FBXO18 | F-box protein, helicase, 18 | Noncitrullinated |
| 163033 | ZNF579 | zinc finger protein 579 | Noncitrullinated |
| 1513 | CTSK | cathepsin K | Noncitrullinated |
| 84196 | USP48 | ubiquitin specific peptidase 48 | Noncitrullinated |
| 64753 | CCDC136 | coiled-coil domain containing 136 | Noncitrullinated |
| 3485 | IGFBP2 | insulin-like growth factor binding protein 2, 36kDa | Noncitrullinated |
| 81551 | STMN4 | stathmin-like 4 | Noncitrullinated |
| 23170 | TTLL12 | tubulin tyrosine ligase-like family member 12 | Noncitrullinated |
| 9454 | HOMER3 | homer homolog 3 (Drosophila) | Noncitrullinated |
| 10144 | FAM13A | family with sequence similarity 13, member A | Noncitrullinated |
| 4858 | NOVA2 | neuro-oncological ventral antigen 2 | Noncitrullinated |
| 708 | C1QBP | complement component 1, q subcomponent binding protein | Noncitrullinated |
| 633 | BGN | biglycan | Noncitrullinated |
| 84081 | NSRP1 | nuclear speckle splicing regulatory protein 1 | Noncitrullinated |
| 6612 | SUMO3 | small ubiquitin-like modifier 3 | Noncitrullinated |
| 7412 | VCAM1 | vascular cell adhesion molecule 1 | Noncitrullinated |
| 1982 | EIF4G2 | eukaryotic translation initiation factor 4 gamma, 2 | Noncitrullinated |
| 9025 | RNF8 | ring finger protein 8, E3 ubiquitin protein ligase | Noncitrullinated |
| 56946 | C11orf30 | chromosome 11 open reading frame 30 | Noncitrullinated |
| 60560 | NAA35 | N(alpha)-acetyltransferase 35, NatC auxiliary subunit | Noncitrullinated |
| 51510 | CHMP5 | charged multivesicular body protein 5 | Noncitrullinated |
| 9796 | PHYHIP | phytanoyl-CoA 2-hydroxylase interacting protein | Noncitrullinated |
| 2629 | GBA | glucosidase, beta, acid | Noncitrullinated |
| 23708 | GSPT2 | G1 to S phase transition 2 | Noncitrullinated |
| 93611 | FBXO44 | F-box protein 44 | Noncitrullinated |
| 80342 | TRAF3IP3 | TRAF3 interacting protein 3 | Noncitrullinated |
| 5455 | POU3F3 | POU class 3 homeobox 3 | Noncitrullinated |
| 116447 | TOP1MT | topoisomerase (DNA) I, mitochondrial | Noncitrullinated |
| 3336 | HSPE1 | heat shock 10kDa protein 1 | Noncitrullinated |
| 684 | BST2 | bone marrow stromal cell antigen 2 | Noncitrullinated |
| 1001 | CDH3 | cadherin 3, type 1, P-cadherin (placental) | Noncitrullinated |
| 4000 | LMNA | lamin A/C | Noncitrullinated |
| 6757 | SSX2 | synovial sarcoma, X breakpoint 2 | Noncitrullinated |
| 3467 | IFNW1 | interferon, omega 1 | Noncitrullinated |
| 3092 | HIP1 | huntingtin interacting protein 1 | Noncitrullinated |
| 83483 | PLVAP | plasmalemma vesicle associated protein | Noncitrullinated |
| 8646 | CHRD | chordin | Noncitrullinated |
| 6628 | SNRPB | small nuclear ribonucleoprotein polypeptides B and B1 | Noncitrullinated |
| 6176 | RPLP1 | ribosomal protein, large, P1 | Noncitrullinated |
| 55827 | DCAF6 | DDB1 and CUL4 associated factor 6 | Noncitrullinated |
| 6897 | TARS | threonyl-tRNA synthetase | Noncitrullinated |
| 2617 | GARS | glycyl-tRNA synthetase | Noncitrullinated |
| 6181 | RPLP2 | ribosomal protein, large, P2 | Noncitrullinated |
| 1107 | CHD3 | chromodomain helicase DNA binding protein 3 | Noncitrullinated |
| 1991 | ELANE | elastase, neutrophil expressed | Noncitrullinated |
| 3035 | HARS | histidyl-tRNA synthetase | Noncitrullinated |
| 4057 | LTF | lactotransferrin | Noncitrullinated |
| 8359 | HIST1H4A | histone cluster 1, H4a | Noncitrullinated |
| 3735 | KARS | lysyl-tRNA synthetase | Noncitrullinated |
| 8349 | HIST2H2BE | histone cluster 2, H2be | Noncitrullinated |
| 6634 | SNRPD3 | small nuclear ribonucleoprotein D3 polypeptide 18kDa | Noncitrullinated |
| 8337 | HIST2H2AA3 | histone cluster 2, H2aa3 | Noncitrullinated |
| 1511 | CTSG | cathepsin G | Noncitrullinated |
| 6738 | TROVE2 | TROVE domain family, member 2 | Noncitrullinated |

| **Supplementary file 1** Overview of measured antigens (*continued*) | | | |
| --- | --- | --- | --- |
| **Gene ID** | **Gene symbol** | **Gene name** | **Type** |
| 1060 | CENPC | centromere protein C | Noncitrullinated |
| 361 | AQP4 | aquaporin 4 | Noncitrullinated |
| 3569 | IL6 | interleukin 6 | Noncitrullinated |
| 16 | AARS | alanyl-tRNA synthetase | Noncitrullinated |
| 6434 | TRA2B | transformer 2 beta homolog (Drosophila) | Noncitrullinated |
| 4599 | MX1 | MX dynamin-like GTPase 1 | Noncitrullinated |
| 7791 | ZYX | zyxin | Noncitrullinated |
| 6737 | TRIM21 | tripartite motif containing 21 | Noncitrullinated |
| 7520 | XRCC5 | X-ray repair complementing defective repair in Chinese hamster cells 5 | Noncitrullinated |
| 80184 | CEP290 | centrosomal protein 290kDa | Noncitrullinated |
| 55727 | BTBD7 | BTB (POZ) domain containing 7 | Noncitrullinated |
| 4670 | HNRNPM | heterogeneous nuclear ribonucleoprotein M | Noncitrullinated |
| 10938 | EHD1 | EH-domain containing 1 | Noncitrullinated |
| 6624 | FSCN1 | fascin actin-bundling protein 1 | Noncitrullinated |
| 6638 | SNRPN | small nuclear ribonucleoprotein polypeptide N | Noncitrullinated |
| 2547 | XRCC6 | X-ray repair complementing defective repair in Chinese hamster cells 6 | Noncitrullinated |
| 337 | APOA4 | apolipoprotein A-IV | Noncitrullinated |
| 6382 | SDC1 | syndecan 1 | Noncitrullinated |
| 5937 | RBMS1 | RNA binding motif, single stranded interacting protein 1 | Noncitrullinated |
| 2023 | ENO1 | enolase 1, (alpha) | Noncitrullinated |
| 4869 | NPM1 | nucleophosmin (nucleolar phosphoprotein B23, numatrin) | Noncitrullinated |
| 29982 | NRBF2 | nuclear receptor binding factor 2 | Noncitrullinated |
| 10134 | BCAP31 | B-cell receptor-associated protein 31 | Noncitrullinated |
| 30011 | SH3KBP1 | SH3-domain kinase binding protein 1 | Noncitrullinated |
| 6629 | SNRPB2 | small nuclear ribonucleoprotein polypeptide B | Noncitrullinated |
| 23299 | BICD2 | bicaudal D homolog 2 (Drosophila) | Noncitrullinated |
| 128866 | ZNF217 | zinc finger protein 217 | Noncitrullinated |
| 1629 | DBT | dihydrolipoamide branched chain transacylase E2 | Noncitrullinated |
| 9091 | PIGQ | phosphatidylinositol glycan anchor biosynthesis, class Q | Noncitrullinated |
| 6729 | SRP54 | signal recognition particle 54kDa | Noncitrullinated |
| 4069 | LYZ | lysozyme | Noncitrullinated |
| 25930 | SRPR | signal recognition particle receptor (docking protein) | Noncitrullinated |
| 6175 | RPLP0 | ribosomal protein, large, P0 | Noncitrullinated |
| 11124 | FAF1 | Fas (TNFRSF6) associated factor 1 | Noncitrullinated |
| 4688 | NCF2 | neutrophil cytosolic factor 2 | Noncitrullinated |
| 350 | APOH | apolipoprotein H (beta-2-glycoprotein I) | Noncitrullinated |
| 90861 | HN1L | hematological and neurological expressed 1-like | Noncitrullinated |
| 23135 | KDM6B | lysine (K)-specific demethylase 6B | Noncitrullinated |
| 23135 | KDM6B | lysine (K)-specific demethylase 6B | Noncitrullinated |
| 7112 | TMPO | thymopoietin | Noncitrullinated |
| 64763 | ZNF574 | zinc finger protein 574 | Noncitrullinated |
| 4841 | NONO | non-POU domain containing, octamer-binding | Noncitrullinated |
| 3181 | HNRNPA2B1 | heterogeneous nuclear ribonucleoprotein A2/B1 | Noncitrullinated |
| 9961 | MVP | major vault protein | Noncitrullinated |
| 3178 | HNRNPA1 | heterogeneous nuclear ribonucleoprotein A1 | Noncitrullinated |
| 4353 | MPO | Myeloperoxidase (MPO; non-recombinant) | Noncitrullinated |
| 6672 | Sp100 | Sp100 | Noncitrullinated |
| 1743 | DLST | OGDC-E2 | Noncitrullinated |
| 1737 | DLAT | PDC-E2 | Noncitrullinated |
| 1108 | Chd4 | Mi-2 | Noncitrullinated |
| 5657 | PRTN3 | Proteinase (PR3; non-recombinant) | Noncitrullinated |
| 5394 | EXOSC10 | PM/Scl 100 | Noncitrullinated |
| 6631 | SNRPC | U1-snRNP C | Noncitrullinated |
| 7150 | TOP1 | DNA Topoisomerase I (Scl-70; truncated) | Noncitrullinated |
| 6741 | SSB | La/SS-B | Noncitrullinated |
| 6634 | SmD3 | SmD3 | Noncitrullinated |
| 6632 | SNRPD1 | SmD1 | Noncitrullinated |
| 6625 | SNRNP70 | U1-snRNP 68/70 kDa | Noncitrullinated |

| **Supplementary file 1** Overview of measured antigens (*continued*) | | | |
| --- | --- | --- | --- |
| **Gene ID** | **Gene ID** | **Gene ID** | **Gene ID** |
| 6626 | SNRPA | U1-snRNP A | Noncitrullinated |
| 7520 | Ku (p70) | Ku (p70) | Noncitrullinated |
| 2547 | Ku (p80) | Ku (p80) | Noncitrullinated |
| 718 | c3 | complement c3 | Noncitrullinated |
